# A Cecal Slurry Mouse Model of Sepsis Leads to Acute Consumption of Vitamin C in the Brain

**DOI:** 10.3390/nu12040911

**Published:** 2020-03-26

**Authors:** David C. Consoli, Jordan J. Jesse, Kelly R. Klimo, Adriana A. Tienda, Nathan D. Putz, Julie A. Bastarache, Fiona E. Harrison

**Affiliations:** 1 Division of Diabetes, Endocrinology, and Metabolism; Vanderbilt University Medical Center, Nashville, TN 37232, USA; david.c.consoli@vanderbilt.edu (D.C.C.); adriana.a.tienda@vumc.org (A.A.T.); 2 Division of Allergy, Pulmonary, and Critical Care Medicine; Vanderbilt University Medical Center, Nashville, TN 37232, USA; jordan.jesse@vumc.org (J.J.J.); nathan.putz@vumc.org (N.D.P.); julie.bastarache@vumc.org (J.A.B.); 3 Undergraduate Program in Neuroscience, Vanderbilt University, Nashville, TN 37232, USA; Kelly.klimo@vanderbilt.edu

**Keywords:** vitamin C, ascorbate, sepsis, brain, cecal slurry, inflammation, cytokines, mouse

## Abstract

Vitamin C (ascorbate, ASC) is a critical antioxidant in the body with specific roles in the brain. Despite a recent interest in vitamin C therapies for critical care medicine, little is known about vitamin C regulation during acute inflammation and critical illnesses such as sepsis. Using a cecal slurry (CS) model of sepsis in mice, we determined ASC and inflammatory changes in the brain following the initial treatment. ASC levels in the brain were acutely decreased by approximately 10% at 4 and 24 h post CS treatment. Changes were accompanied by a robust increase in liver ASC levels of up to 50%, indicating upregulation of synthesis beginning at 4 h and persisting up to 7 days post CS treatment. Several key cytokines interleukin 6 (IL-6), interleukin 1β (IL-1β), tumor necrosis factor alpha (TNFα), and chemokine (C-X-C motif) ligand 1 (CXCL1, KC/Gro) were also significantly elevated in the cortex at 4 h post CS treatment, although these levels returned to normal by 48 h. These data strongly suggest that ASC reserves are directly challenged throughout illness and recovery from sepsis. Given the timescale of this response, decreases in cortical ASC are likely driven by hyper-acute neuroinflammatory processes. However, future studies are required to confirm this relationship and to investigate how this deficiency may subsequently impact neuroinflammation.

## 1. Introduction

Sepsis is estimated to affect more than 30 million people and account for more than 5 million deaths annually [1]. During this critical illness, the robust inflammatory response of the immune system includes release of multiple cytokines and other signaling molecules that can ultimately lead to severe tissue injury and multiple organ failure [2,3,4]. This damage extends to the brain as evidenced by delirium in the majority of patients [5]. Despite the advances in clinical understanding of delirium during sepsis, little is known about the cellular and molecular underpinnings of acute brain dysfunction in critically ill patients. Sepsis patients often exhibit low plasma levels of vitamin C (ascorbate, ASC) [6,7,8,9]. In one study, as many as 88% of sepsis patients had subnormal plasma levels of ASC (<23 µM) and up to 38% had severe ASC deficiency (<11 µM) [10], suggesting high demand for ASC during septic insult. Lower plasma levels of ASC are also associated with increased incidence of multiple organ failure and decreased survival [11]. The brain is particularly susceptible to this dysregulated inflammatory response and to suboptimal ASC levels, because higher oxidative stress levels in the brain are especially damaging to its enriched lipid composition and nutrient-demanding metabolic rate [12,13]. Up to 30% of patients are reported to experience cognitive deficits following recovery from sepsis [14,15,16], and several studies using rodent sepsis models have shown that acute illness damages cognition in surviving animals [17,18,19].

ASC is a critical antioxidant for cellular function and has an emerging role in immune function [20]. Preclinical studies have shown a variety of beneficial effects on the pathophysiological changes in sepsis, including protection against microvascular dysfunction and deficits in vasoconstriction [21,22,23] by preserving tight endothelial barrier function and capillary blood flow [24,25,26]. ASC administration during sepsis also attenuates acute lung injury [27] and improves multiple organ dysfunction syndrome in animal models of sepsis [28,29]. The role of intravenous ASC in clinical practice to improve short term patient recovery is still under clinical investigation [30,31]. ASC accumulates to high levels in the brain via the sodium-dependent vitamin C transporter 2 (SVCT2) in a two-step, energy-dependent process from blood into the choroid plexus cerebral spinal fluid and then into neurons [32]. In the brain, ASC serves two primary roles as a neuroprotector and neuromodulator [32,33,34]. ASC maintains blood–brain barrier integrity by preserving tight endothelial barriers [35,36] and maintaining capillary blood flow [37]. ASC is a critical enzymatic co-factor in neurotransmitter synthesis and DNA methylation [32,38], and is intimately involved in preserving glutamatergic neurotransmission through the glutamate-uptake ASC-release exchange [39,40,41]. Despite the interest in ASC as a treatment for preventing organ failure in sepsis, the roles of ASC in the brain, and the number of patients experiencing cognitive deficits following recovery from the acute trauma, the effects of sepsis on the brain are understudied. Furthermore, the specific role of ascorbate in sepsis-induced brain dysfunction has not been studied. Here, we utilized a cecal slurry (CS) model of sepsis in mice to observe changes in ASC and inflammatory response in the brain during and following sepsis. We hypothesized that ASC is depleted in sepsis, and sought to define a timeline for changes in ASC level and cytokine release, particularly in the brain.

## 2. Materials and Methods

### 2.1. Mouse CS or LPS Treatment

All experiments conducted with live mice were reviewed and approved by the Vanderbilt Institutional Animal Care and Use Committee. Cecal slurry (CS) was used to induce acute peritonitis in mice as a model of sepsis as previously described [42,43,44]. C57/Bl6J donor mice at six weeks of age were obtained from Jackson Laboratory (#000664) and euthanized within 7 days of arrival. Cecal contents were collected and resuspended in 5% dextrose at 80 mg/mL, then filtered through a 100 µm filter. Aliquots were stored at -80 °C until ready for use.

All mice for treatment groups were bred in house from C57/Bl6J mice originally obtained from Jackson Laboratory. Mice at 10–12 weeks of age were treated with CS (1.5 mg/g; i.p.) or the vehicle 5% dextrose for control groups. Another widely used model of peripheral inflammatory response utilizes lipopolysaccharide (LPS) administration. For LPS studies, mice at 6–8 weeks of age received LPS (3.75 µg/g; i.p.) or saline. Control and treated mice were distributed across cages and provided with supplemental nutrition on the floor of the cage (DietGel 76A, Clear H_2_O) to promote survival.

### 2.2. Evaluation of Sickness Score

An observer blinded to treatment groups scored the mice on the severity of sepsis using a 12-point scale of sickness severity, where 12 is healthy with normal activity and 0 is moribund [45,46]. In brief, the score is determined by response to finger poke (4 for normal, 3 for decreased, 2 for severely decreased, or 1 for minimal response), signs of encephalopathy (4 for normal, 3 for tremors or staggering, 2 for twisting movements, or 1 for turning), and general appearance (score is decreased by 1 for each display of piloerection, periorbital exudates, respiratory distress, or diarrhea). All mice were monitored closely for 48 h or until mice recovered to a normal score of 12. CS-treated mice that never received a score below 10 or died prior to the assigned timepoint were excluded from analysis.

### 2.3. Tissue Collection

Mice were anesthetized with isoflurane then euthanized by decapitation at 4, 24, 48 h or 7 days post-injection. Control mice were euthanized at each timepoint and data collapsed into one group. Tissue samples were collected, flash-frozen on dry ice, and stored at –80 °C for further analysis.

### 2.4. Ascorbic Acid HPLC

Sample extracts were prepared by adding 10 µl extraction buffer (7:2 25% *w/v* metaphosphoric acid: 100 mM sodium phosphate, 0.05 mM EDTA pH 8.0) per mg of wet tissue to normalize by weight. Samples were homogenized with 0.5 mm ceria-stabilized zirconium oxide beads (Next Advance, Inc.) in a bullet homogenizer, centrifuged at 10,000 rpm, and the clear supernatant transferred into a fresh tube. Concentrations of ASC were measured at 1:100 dilution in triplicate with ion pair HPLC, using tetrapentyl ammonium bromide as the ion pair reagent and electrochemical detection as previously described [47,48].

### 2.5. Determination of Gene Expression

RNA was extracted using an RNeasy Mini Kit (Qiagen). qPCR was performed using a PrimePCR Probe Assay consisting of iScript cDNA synthesis, PrimePCR Probes (PrimePCR™ Probe Assay: Slc23a1, Mouse), and Sso Advanced Universal Supermix (Bio Rad).

### 2.6. Measurement of Oxidative Stress Markers

Malondialdehyde, a lipid peroxidation end product, was measured by fluorescent spectrophotometric assay of thiobarbituric acid reactive substances (TBARS) as previously described [49]. Sulfhydryls were measured by reduction of 5,5’-dithiobis (2-nitrobenzoic acid) (DTNB) to 2-nitro-5-thiobenzoate (TNB) anion by thiol groups and spectrophotometric analysis [50,51].

### 2.7. Measurement of Cytokine Expression

Frozen mouse tissues were homogenized in volumes of RIPA buffer (Thermo Fisher Scientific) normalized by tissue weight. Tissue levels of IFN-y, IL-1b, IL-6, KC/GRO, IL-10, and TNF-alpha were assayed in duplicate using a V-PLEX Custom Mouse Cytokine Kit, (Meso Scale Diagnostics, LLC) according to the manufacturer’s instructions [52].

### 2.8. Statistical Analyses

Statistical analyses were performed using Graphpad Prism software (version 8.3.0). Data were first checked for equality of group variances using the Brown–Forsyth test and analyzed using parametric statistics. We did not expect any differences in response to CS according to sex, and all data were first analyzed using a multivariate ANOVA analysis, including sex as an additional variable. There were no main effects of sex on any of the key outcomes (sickness score, weight loss, ASC levels) so data were combined for all subsequent analysis. For outcomes following CS treatment, data were analyzed with univariate ANOVA with group (encompassing treatments and time post treatment) as the main independent variable. Significant omnibus ANOVA were followed with Dunnett’s post hoc analyses to test difference from the control group. Independent t-tests were used to test effects of LPS versus treatment with the vehicle. Numerical outliers that likely reflected experimental error were identified and removed using ROUT (Q = 5%). Error bars are shown as SEM or SD as indicated in figure legends.

## 3. Results

### 3.1. Cecal Slurry Treatment Induces Acute Peritonitis and Weight Loss

**Figure 1:**
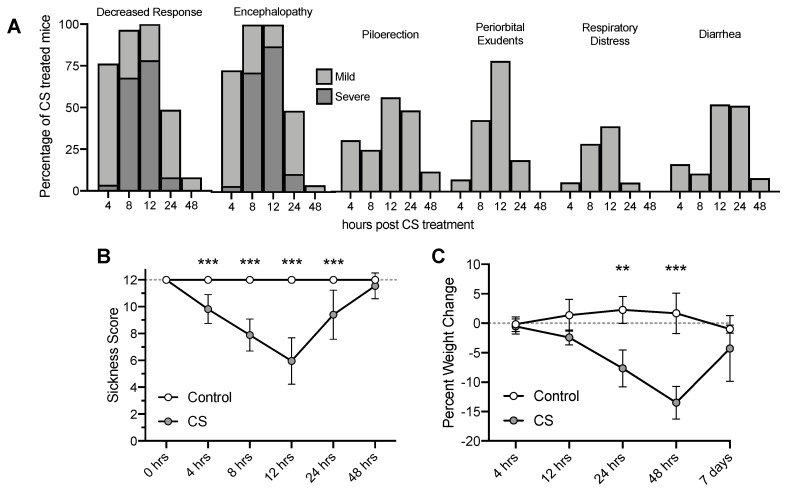
Observed sickness scores of mice and percent weight change over time following Cecal Slurry (CS) treatment. (**A**) Percentage of mice showing sickness behaviors and appearances. (**B**) Clinical Sickness Scores. (**C**) Weight loss. ** p < 0.01*** *p* < 0.001. Error bars plotted as mean ± SD.

Mice that received cecal slurry (CS) treatment became severely lethargic and exhibited decreased responses to stimuli, signs of encephalopathy, and worsened appearance by 12 h (Figure 1A). Sickness scores decreased as early as 4 h, began to recover by 24 h, and returned to normal appearance and activity by 48 h (Figure 1B; CS Treatment F_(1, 63)_ = 113.8, *p* < 0.001; Time F_(5, 59)_ = 15.61, *p* < 0.001). CS treatment also caused significant weight loss that persisted to 48 h, although mice regained weight by 7 days post CS treatment (Figure 1C; CS Treatment F_(1, 63)_ = 121.3, *p* < 0.001; Time F_(5, 59)_ = 24.06, *p* < 0.001). Out of 55 CS treated mice, only two mice died before their scheduled timepoint. This mild 1.5 mg/g dose was chosen to optimize survival to 7 days post CS treatment. The 4% mortality rate at this dose is low compared to other studies utilizing this CS model (2.0 mg/g, up to 67% by 48 h) [42,44].

### 3.2. Tissue ASC Concentrations Following CS Treatment

**Figure 2:**
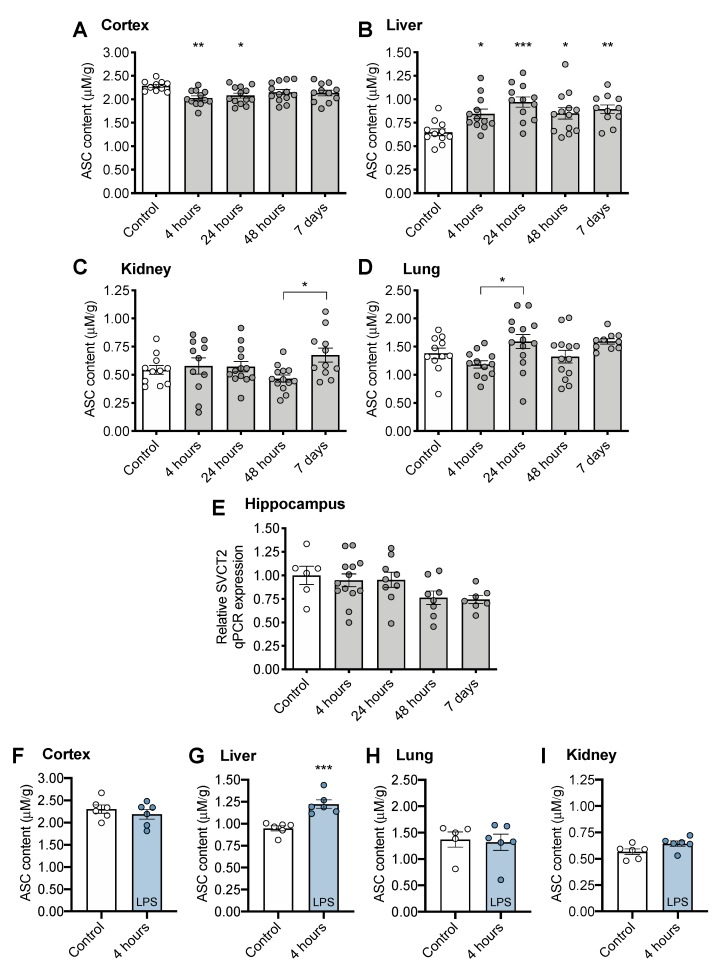
Tissue ASC (ascorbate) concentrations following CS treatment in (**A**) cortex, (**B**) liver, (**C**) kidney, (**D**) lung. (**E**) Sodium-dependent vitamin C transporter 2, SVCT2 gene expression in brain following CS treatment. Tissue ASC concentrations following LPS treatment (**F**) brain, (**G**) liver, (**H**) lung, and (**I**) kidney. * p < 0.05 ** p < 0.01 *** *p* < 0.001 from control following significant ANOVA results unless otherwise indicated. Error bars plotted as mean ± SEM.

There was a small (~10% at 4 h) but significant decrease in cortical ASC at 4 and 24 h following CS treatment compared to controls (Figure 2A, F_(4, 54)_ = 3.216, *p* = 0.0193,), indicating consumption of brain ASC reserves. Liver ASC levels were significantly increased following CS treatment (~50% at 24 h) indicating robust upregulation of ASC synthesis (Figure 2B, F_(4, 54)_ = 5.090, *p* = 0.0015) that persisted to 7 days post CS treatment. No significant differences in ASC levels were observed in peripheral organs of CS-treated mice compared to controls, although levels varied post CS treatment (kidney: Figure 2C, F_(4, 54)_ = 2.153, *p* = 0.0867; lung: Figure 2D, F_(4, 55)_ = 3.188, *p* = 0.020). We hypothesized that a decrease in ASC levels in the brain would result in upregulation of SVCT2 expression, though no significant changes were observed in hippocampal SVCT2 expression in response to CS treatment (Figure 2E, F_(4, 38)_ = 2.235, *p* = 0.0833).

No decrease in brain ASC level was observed at 4 h following LPS treatment (Figure 2F, t(10) = 0.842, *p* = 0.4197). However, LPS treatment also induced upregulation of ASC synthesis in liver (Figure 2G, t(10) = 4.913, *p* < 0.001). No changes were observed in lung (Figure 2H, t(9) = 0.246, *p* = 0.8111), or kidney (Figure 2I, t(10) = 2.066, *p* = 0.0658).

### 3.3. CS Treatment Does Not Induce Changes in Oxidative Stress Measurements

**Figure 3:**
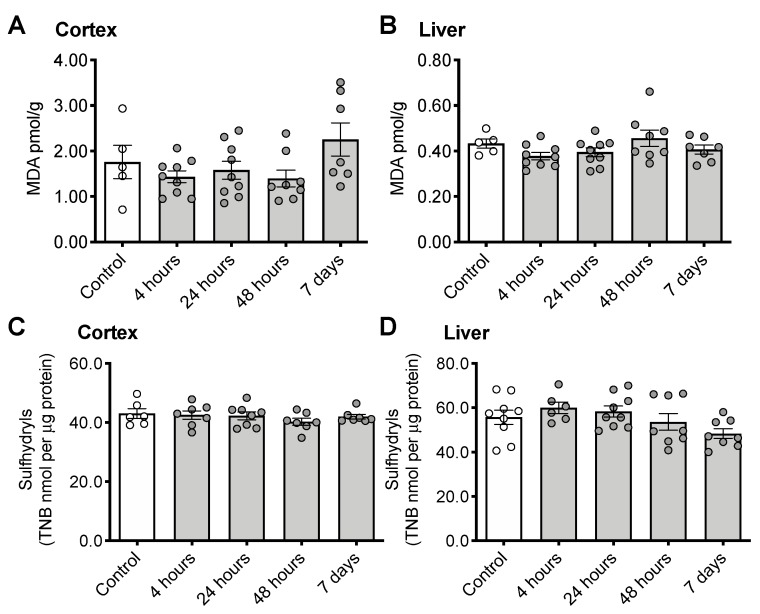
Indicators of oxidative stress following CS treatment. Malondialdehyde (MDA) in cortex (**A**) and liver (**B**) or sulfhydryls in cortex (**C**) and liver (**D**). Error bars plotted as mean ± SEM.

CS treatment did not increase either of the oxidative stress markers malondialdehyde (cortex: Figure 3A, F_(4, 33)_ = 2.092, *p* = 0.1042; Liver: Figure 3B, F_(4, 33)_ = 1.833, *p* = 0.1459) or sulfhydryls (cortex: Figure 3C, F_(4, 30)_ = 0.6471, *p* = 0.6333; Liver: Figure 3D, F_(4, 35)_ = 2.210, *p* = 0.0880) in the brain.

### 3.4. CS Treatment Initiates an Inflammatory Response in the Brain

**Figure 4:**
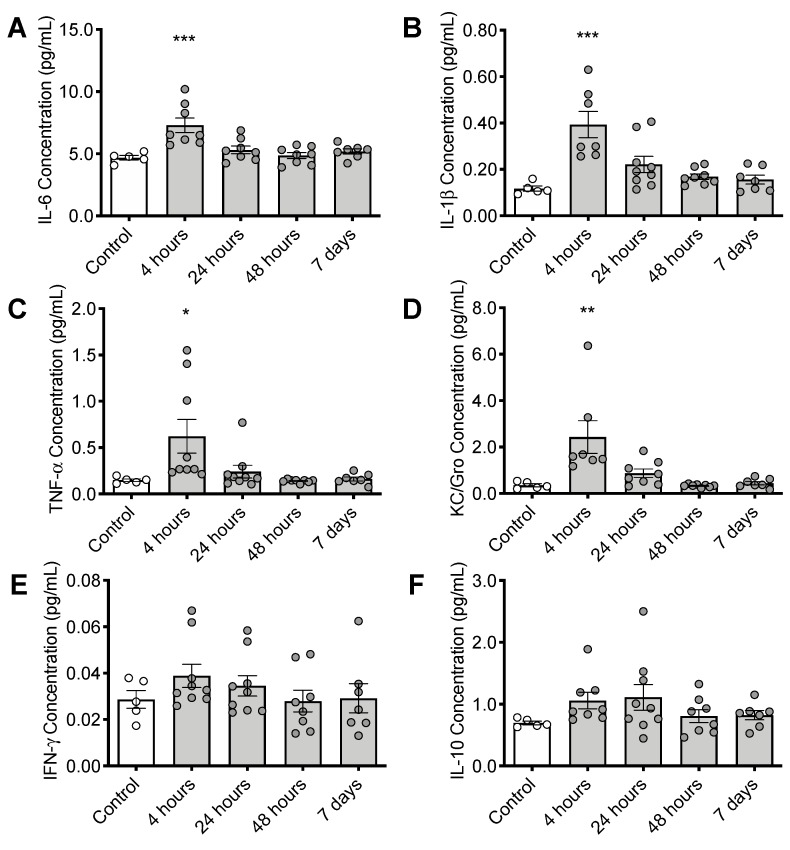
Timeline of inflammatory changes in the brain following CS treatment. Cortical cytokine levels of (**A**) interleukin 6 (IL-6), (**B**) interleukin 1β (IL-1β), (**C**) tumor necrosis factor alpha (TNFα), and (**D**) chemokine (C-X-C motif) ligand 1 (CXCL1, KC/Gro), (**E**) Interferon gamma (INFγ) and (**F**) interleukin 10 (IL-10) * p < 0.05, ** p < 0.01, *** *p* < 0.001. Error bars plotted as mean ± SEM.

In cortex, expression of several proinflammatory cytokines was elevated at 4 h post CS treatment including interleukin 6 (IL-6: F_(4, 31)_ = 8.356, *p* < 0.001), interleukin 1β (IL-1β: F_(4, 31)_ = 9.742, *p* < 0.001), tumor necrosis factor alpha (TNFα: F_(4, 33)_ = 4.261, *p* = 0.0069), and chemokine (C-X-C motif) ligand 1 (CXCL1, KC/Gro: F_(4, 30)_ = 7.091, *p* < 0.001) (Figure 4A–D). Cytokine expression levels returned to normal by 48 h post CS treatment. More modest increases in interferon gamma (INFγ: F_(4, 33)_ = 0.9214, *p* = 0.4632) and interleukin 10 (IL-10: F_(4, 32)_ = 1.442, *p* = 0.2430) were not statistically significant (Figure 2E,F).

## 4. Discussion

Despite significant clinical interest in ASC as a potential therapeutic adjuvant, the role of ASC in sepsis, particularly in the brain, has not been well studied. The present study highlights the potential rapid, although modest, consumption of ASC stores during and following sepsis co-occurring with the associated inflammatory changes that occur in the brain.

Mice and most other rodent species possess the gene encoding gulonolactone oxidase, an enzyme responsible for catalyzing the final step in ASC synthesis in the liver [53,54,55,56]. Synthesis can be upregulated to provide higher tissue levels in the liver under periods of increased physiological need, such as pregnancy [57]. ASC depletion in the brain is rare in non-genetically modified mice, however, increased liver levels indicate upregulation of ASC synthesis to prevent depletion. In the brain, high concentrations are critical for maintaining optimal brain function and preventing oxidative damage [32,33,58]. CS treatment resulted in an approximately 10% decrease in ASC in cortex (Figure 2A) even in this relatively mild sepsis model, and despite upregulation of synthesis in the liver. The two-step transport system of ASC into the brain by SVCT2 from blood into the choroid plexus cerebral spinal fluid and then into neurons allows for preservation of ASC at the expense of other tissues [32]. It is possible that other brain regions, such as the hippocampus, may have different levels of susceptibility to ASC depletion due to varying proximity to the ventricles and choroid plexus, and future studies should address different brain areas, including hippocampus, in analysis.

Over this short time interval, we did not observe decreased ASC levels in most peripheral organs including lung and kidney (Figure 2C,D) and heart, muscle, spleen, and adrenal gland (data not shown). We observed a significant increase in liver ASC by 4 h in both CS- and LPS-treated mice (Figure 2B,G). Our data confirm results of prior work in which an LPS-induced increase in liver ASC was observed from 3 h post LPS treatment [59]. LPS treatment did not fully recapitulate the ASC changes we observed with CS treatment, possibly due to the dose initiating a lower neuroinflammatory effect or different pathways, although this was not directly tested. While no changes were observed in brain in that study [59], the lack of cortical ASC deficiency in the LPS model may be a limitation of intraperitoneal LPS as a model of sepsis. We hypothesize that increased synthesis in the liver provided sufficient circulating levels to replenish (brain) or protect (lung, kidneys) peripheral tissues during the time period and for the dose studied. It took more than 24 h to replenish brain ASC, possibly due to slower brain uptake, since ASC must first accumulate in the cerebral spinal fluid. Future experiments should be performed in genetically modified mice that are, like humans, incapable of synthesizing ASC (e.g., gulonolactone oxidase knockout mice) [47,60], and are therefore unable to upregulate hepatic synthesis to meet increased need. Such models could also be used to establish a timeline of circulating ASC levels in plasma as well as testing whether a clinically relevant ASC pretreatment [61] is capable of protecting against decreased brain ASC, neuroinflammatory changes, or severity of observed sickness. If peripheral ASC administration is unable to change cortical ASC levels, this would suggest blood–brain barrier transport of ASC is limiting early in sepsis or that there is high uptake by other organs. However, if neuroinflammation is improved by pretreatment with ASC, this would suggest that ASC deficiency may be directly associated with sepsis-associated neuroinflammation.

The CS dosing regimen used (1.5 mg/g) causes a mild sickness response, chosen to optimize recovery and survival to 7 days post treatment. Nevertheless, this modest insult was still sufficient to deplete ASC and increase cytokine production in the brain at 4 h after injection (Figure 4), when the mice are just starting to become systemically ill. This finding suggests that the brain is adversely affected by systemic inflammation in the earliest stages of illness. The release of cytokines in the brain at this time indicates activation of brain macrophages and resident microglia, which generate reactive oxygen species. The observed ASC depletion likely indicates ASC plays a primary role in electron donation to neutralize these free radicals, and that the oxidative stress was sufficient to overwhelm ASC recycling capacity. While future studies will need to clarify the relationship between ASC deficiency and neuroinflammation, it is most likely that ASC deficiency is driven by the acute neuroinflammatory response and associated generation of radical species. Oxidative stress is a key component of clinical sepsis [62], and although the global oxidative measurements did not show elevations in brain or liver oxidative stress under these conditions, any reduction in brain ASC and especially upregulation of liver ASC synthesis indicates elevated oxidative challenge. The measures of global tissue MDA may not have been sensitive enough to detect localized increases in oxidative stress in specific cells (endothelium, for example) or tissue compartments. Despite the activated immune response and challenge of brain ASC stores, we did not observe upregulation of the sodium-dependent vitamin C transporter SVCT2 in brain tissue. To confirm the association between ASC consumption and acute inflammatory challenge in a second model, we used LPS to induce endotoxemia and systemic inflammation. LPS treatment was also sufficient to upregulate liver ASC synthesis by 4 h. Whether higher doses of CS or LPS would cause further or prolonged depletion of brain ASC in a more severe illness model should be studied in future experiments.

ASC deficiency is well defined in critical care patient populations [6,7,8,9,10]. Many preclinical studies have shown that early ASC supplementation or IV treatment can protect against vascular and organ dysfunctions associated with sepsis [22,23,26]. One possible explanation for the potential beneficial effects of ASC is that hospitalized patients who develop sepsis are more likely to have underlying ASC deficiency, either as a result of chronic illness, co-morbidities, or poor diet. However, our results and many others suggest that ASC depletion is directly caused by the illness itself, likely due to massive inflammatory challenge, endothelial breakdown, and elevated oxidative stress [35,37]. Although some clinical studies have associated lower plasma levels of ASC with increased incidence of multiple organ failure and decreased survival [11], several phase I clinical studies have shown that IV administration of ASC during sepsis does not improve or worsen short term survival outcomes [8,31,63]. Overall survivability during sepsis is dependent on a variety of factors including antibiotics administration, prior health status, prior injury or illness, and age [64]. While maintenance of ASC levels during sepsis may not directly impact acute survival, it may be critical to protection against inflammatory damage following sepsis, especially in the brain [14]. Future studies will seek to understand how ASC is involved in the acute inflammatory response and the implications for long term cognitive dysfunction following recovery.
